# Role of Glucose Metabolic Reprogramming in Breast Cancer Progression and Drug Resistance

**DOI:** 10.3390/cancers15133390

**Published:** 2023-06-28

**Authors:** Pan Lei, Wenzhou Wang, Marisela Sheldon, Yutong Sun, Fan Yao, Li Ma

**Affiliations:** 1 Hubei Hongshan Laboratory, College of Biomedicine and Health, Huazhong Agricultural University, Wuhan 430070, China; leipanws@webmail.hzau.edu.cn (P.L.); wangwenzhou@webmail.hzau.edu.cn (W.W.); 2 Hubei Key Laboratory of Embryonic Stem Cell Research, School of Basic Medical Sciences, Taihe Hospital, Hubei University of Medicine, Shiyan 442000, China; 3 Department of Experimental Radiation Oncology, The University of Texas MD Anderson Cancer Center, Houston, TX 77030, USA; mesheldon@mdanderson.org; 4 Department of Molecular and Cellular Oncology, The University of Texas MD Anderson Cancer Center, Houston, TX 77030, USA; ysun2@mdanderson.org; 5 College of Life Science and Technology, Huazhong Agricultural University, Wuhan 430070, China; 6 Shenzhen Institute of Nutrition and Health, Huazhong Agricultural University, Shenzhen 518000, China; 7 Shenzhen Branch, Guangdong Laboratory for Lingnan Modern Agriculture, Genome Analysis Laboratory of the Ministry of Agriculture, Agricultural Genomics Institute at Shenzhen, Chinese Academy of Agricultural Sciences, Shenzhen 518000, China; 8 The University of Texas MD Anderson Cancer Center UTHealth Houston Graduate School of Biomedical Sciences, Houston TX 77030, USA

**Keywords:** breast cancer, glucose metabolism, metastasis, drug resistance, Warburg effect, reverse Warburg effect, tumor microenvironment, metabolic inhibitor

## Abstract

**Simple Summary:**

Glucose metabolic reprogramming is a process in which cells alter the way that glucose is utilized. In breast cancer, cells can undergo this process to fuel their growth, acquire drug resistance, and become metastatic. Scientists have been studying this process to identify new targets for breast cancer treatment. By understanding what enzymes and pathways are involved, researchers may be able to develop drugs that specifically target these pathways, ultimately leading to more effective and less toxic treatments for breast cancer in the future.

**Abstract:**

The involvement of glucose metabolic reprogramming in breast cancer progression, metastasis, and therapy resistance has been increasingly appreciated. Studies in recent years have revealed molecular mechanisms by which glucose metabolic reprogramming regulates breast cancer. To date, despite a few metabolism-based drugs being tested in or en route to clinical trials, no drugs targeting glucose metabolism pathways have yet been approved to treat breast cancer. Here, we review the roles and mechanisms of action of glucose metabolic reprogramming in breast cancer progression and drug resistance. In addition, we summarize the currently available metabolic inhibitors targeting glucose metabolism and discuss the challenges and opportunities in targeting this pathway for breast cancer treatment.

## 1. Introduction

**Figure 1:**
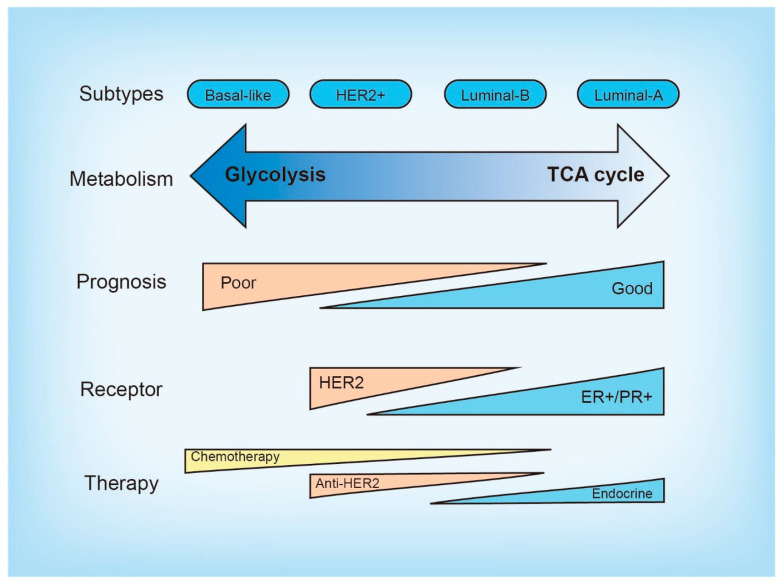
Characteristics of breast cancer subtypes. Different subtypes of breast cancer vary in terms of metabolism, prognosis, receptor expression, and treatments. The luminal-A and luminal-B subtypes tend to undergo the tricarboxylic acid cycle (TCA cycle) and oxidative phosphorylation, while the basal-like and HER2+ subtypes tend to undergo glycolysis.

Female breast cancer is the most common cancer type worldwide, accounting for 24.5% of cancer incidences and 15.5% of cancer deaths in women [1]. Based on the presence or absence of clinical biomarkers, breast cancer can be divided into three subtypes: hormone receptor-positive, HER2-positive, and triple-negative (ER^−^, PR^−^, HER2^−^) [2]. Among all cases, approximately 70% are hormone receptor-positive, while HER2-positive or triple-negative breast cancer each accounts for approximately 15% [2]. Based on the molecular classification, breast cancer is categorized into four subtypes: luminal A, luminal B, HER-2 enriched, and basal-like (Figure 1) [3,4]. Whereas 50–75% of TNBC patients are basal-like [5], luminal-A and luminal-B subtypes overlap with ER^+^HER2^−^ and ER^+^HER2^+^ subtypes, respectively [6], although a fraction of luminal-B subtypes is HER2^−^ [7]. Current options for systemic breast cancer therapies include chemotherapy, CDK4/6 inhibitors, endocrine therapy, and HER2-targeting drugs [2,8,9,10]. Some cases are intrinsically resistant to treatment, and among the patients who show an initial response, acquired resistance is common. Ultimately, de novo or acquired therapy resistance results in local and metastatic recurrences and death.

Alterations in glucose metabolism are common metabolic changes that distinguish tumor cells from normal cells. The changes in glucose metabolism, first discovered in the 1920s by Otto Warburg, are characterized by the tendency of cancer cells to undergo glycolysis to produce lactate, despite oxygen-enriched conditions. This is known as the “Warburg effect” [11]. A “reverse Warburg effect” theory describes that cancer cells promote aerobic glycolysis in tumor-associated fibroblasts and that lactate and pyruvate secreted by these cells, in turn, promote tumor growth and progression [12]. Besides glycolysis, glucose is also utilized by other metabolic pathways, including the hexosamine pathway, the pentose phosphate pathway, and the serine biosynthesis pathway [13]. Rewiring of glucose metabolism and increased glycolytic flux in tumor cells are associated with de novo or acquired resistance to anticancer drugs [14,15]. Moreover, metabolic reprogramming is linked to cancer cell migration and metastasis [16]. The earliest drug to target metabolism was aminopterin, which was found to inhibit folic acid synthesis and induce remissions in childhood acute lymphocytic leukemia [15]. Subsequently, other anti-metabolite drugs, including those targeting nucleotide metabolism, have been approved for clinical use. Notably, the approval of drugs targeting mutant isocitrate dehydrogenases to treat acute myeloid leukemia represents a landmark in targeting cancer metabolism in the precision medicine era [15].

## 2. Molecular and Metabolic Heterogeneity in Breast Cancer

Breast cancer is characterized by molecular and metabolic heterogeneity. The luminal-A subtype tends to exhibit the reverse Warburg effect, whereas TNBC is associated with Warburg and mixed types [17]. Heterogeneous molecular characteristics of breast cancer subtypes underlie metabolic heterogeneity. For instance, the frequency of p53 mutations is much higher in basal-like and HER2-enriched subtypes than in the luminal subtypes [18]. Wild-type p53 blocks glycolytic flux and promotes oxidative phosphorylation (OXPHOS) by downregulating glycolytic enzymes and upregulating mitochondrial proteins [19], and p53-deficient cancer cells tend to undergo metabolic shifts toward glycolysis [20,21]. Moreover, basal-like breast cancer often expresses high levels of c-Myc and EGF, and ER-positive breast cancer frequently harbors activating mutations of PI3K. c-Myc, EGFR signaling, and the activated PI3K pathway all promote breast cancer aerobic glycolysis, suggesting the roles of these pathways in the metabolic type of these breast cancer subtypes [22,23,24].

## 3. Reprogramming of Glucose Metabolism in Breast Cancer Progression and Therapy Response

In this section, we discuss the implications of key factors of glucose metabolism, including hexokinase (HK), phosphofructokinase (PFK), pyruvate kinase (PK), pyruvate dehydrogenase kinase (PDK), 3-phosphoglycerate dehydrogenase (PHGDH), and lactate dehydrogenase (LDH), in breast cancer progression and drug resistance (Table 1).

### 3.1. Hexokinase (HK)

**Figure 2:**
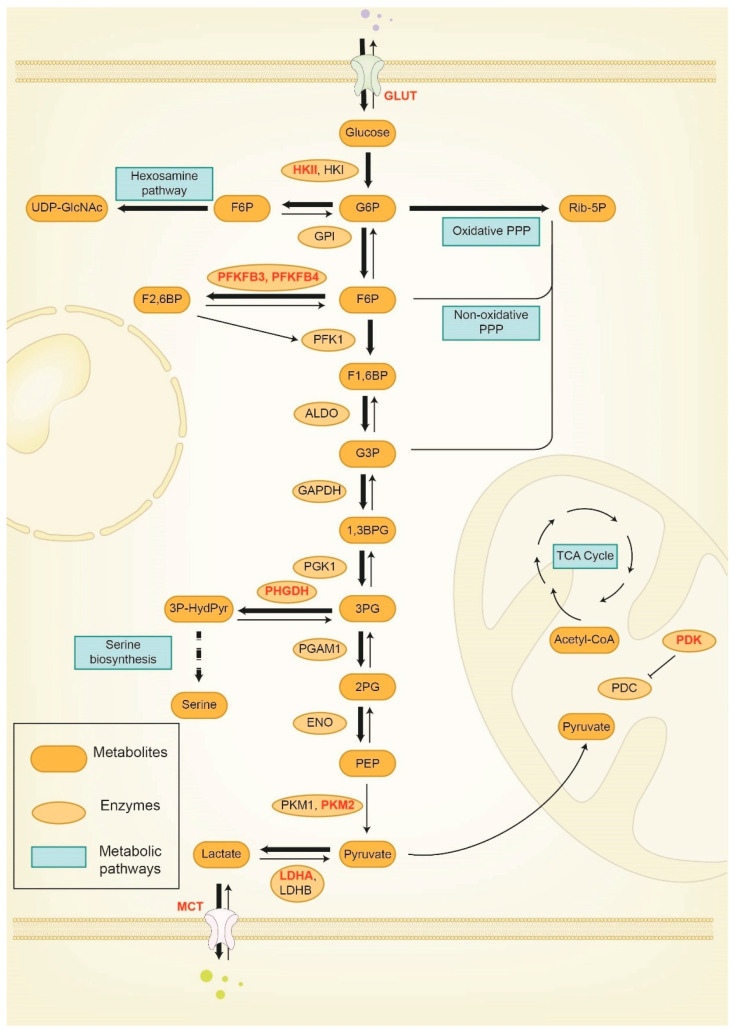
Glucose metabolism in cancer cells. The flux of glucose metabolism in cancer cells is characterized by an increase in glucose uptake and aerobic glycolysis, involving a series of reactions that convert glucose into pyruvate. The red color indicates enzymes that are overexpressed in breast cancer cells. The thick arrow indicates the favored flux in breast cancer cells. The thin arrow indicates the unfavored flux in breast cancer cells. The curved arrows indicate the TCA cycle. The dotted arrow indicates the serine biosynthesis pathway. HK: hexokinase; G6P: glucose 6-phosphate; F6P: fructose 6-phosphate; UDP-GlcNAc: uridine diphosphate N-acetylglucosamine; GPI: glucose-6-phosphate isomerase; Rib-5P: ribofuranose 5-phosphate; PFK: phosphofructokinase; F1,6BP: fructose 1,6-bisphosphate; F2,6BP: fructose 2,6-bisphosphate; G3P: glyceraldehyde 3-phosphate; GAPDH: glyceraldehyde 3-phosphate dehydrogenase; 1,3BPG: 1,3-bisphosphoglycerate; PGK: phosphoglycerate kinase; 3PG: 3-phosphoglyceric acid; PHGDH: phosphoglycerate dehydrogenase; PGAM: phosphoglycerate mutase; 2PG: 2-phosphoglycerate; ENO: enolase; PEP: polyestradiol phosphate; PK: pyruvate kinase; LDH: lactate dehydrogenase; 3P-HydPyr: 3-phosphonooxypyruvate; PDC: pyruvate dehydrogenase complex; PDK: pyruvate dehydrogenase kinase; TCA cycle: tricarboxylic acid cycle; PPP: pentose phosphate pathway.

HK, the first rate-limiting enzyme in glycolysis, catalyzes the phosphorylation of glucose to glucose-6-phosphate (G6P), which maintains a glucose gradient promoting its uptake (Figure 2) [25]. There are four isoforms of HK, namely HKI, HKII, HKIII, and HKIV. Because HKI, HKII, and HKIII have a high affinity with G6P, G6P exerts a feedback inhibition on these kinases, but not on HKIV due to its low affinity with G6P. Mammalian HKI is expressed in all tissues and is known as the “housekeeping enzyme” [26]. HKIII is poorly expressed in most tissues; whether and how it regulates breast cancer remains unclear [27]. HKII is highly expressed in embryonic tissues as well as adult adipose and muscle tissues. Interestingly, HKII is also expressed in breast cancer cells and promotes glycolysis and glucose uptake. In breast cancer, HKII can be upregulated by transcriptional activation, post-translational modifications (PTMs), and non-coding RNAs. miR-155, an inflammation-induced microRNA (miRNA), upregulates HKII to promote glycolysis in breast cancer cells [28]. Mechanistically, miR-155 targets C/EBPβ, resulting in the downregulation of miR-143, a miRNA that targets HKII. Certain circular RNAs have been shown to promote glycolysis in breast cancer cells by upregulating HKII. For example, circRNF20 sponges miR-487a, a miRNA targeting HIF-1α, leading to HIF-1α upregulation. HIF-1α, in turn, binds to the HKII gene promoter and promotes its transcription [29]. Among glucose metabolism-regulating PTMs, HECTH9-mediated K63-linked ubiquitination of HKII promotes its mitochondrial translocation, upregulates its activity, and enhances glycolysis in breast cancer cells [30]. Moreover, PIM2-mediated Thr473 phosphorylation of HKII promotes breast cancer cell glycolysis and autophagy under glucose starvation conditions [31].

A growing body of evidence has demonstrated that HKII plays a functional role in breast cancer progression and correlates with metastasis [32,33]. By using MMTV-Neu-IRES-Cre and HKII conditional knockout mouse models, Hay and colleagues showed that knockout of HKII in mice suppressed oncogene-induced mammary tumor growth [32]. Moreover, knockout of HKII inhibited lung metastasis in an MMTV-PyMT mouse model of metastatic breast cancer, and knockdown of HKII in the 4T1 mouse mammary tumor cell line blocked its lung-metastatic ability [34]. Mechanistic studies revealed that HKII can act as an anchoring protein to form a ternary complex with the regulatory subunit of protein kinase A and GSK3β, which promotes phosphorylation of GSK3β, leading to decreased GSK3β activity and elevated stability of GSK3 targets, such as Snail. In addition, the kinase activity of HKII promotes glycosylation of Snail, which prevents it from GSK3β-dependent phosphorylation and degradation, thereby inducing Snail-mediated epithelial-mesenchymal transition and metastasis in breast cancer [34]. In addition, HKII is involved in breast cancer therapy resistance. For instance, HKII promotes autophagy by inhibiting the mTOR-S6K pathway, leading to tamoxifen resistance in the MCF-7 human breast cancer cell line [35]. Furthermore, phosphorylation of HKII by PIM2 promotes glycolysis and autophagy, which confers breast cancer resistance to paclitaxel [31]. Taken together, these findings suggest that HKII is a potential therapeutic target for breast cancer treatment.

### 3.2. Phosphofructokinase (PFK)

Phosphofructokinase-1 (PFK-1), the second rate-limiting enzyme in glycolysis, catalyzes the conversion of fructose-6-phosphate to fructose-1,6-bisphosphate (Figure 2). Human PFK-1 has three isoforms: PFKL, PFKM, and PFKP. Whereas muscle tissues exclusively express PFKM, other tissues may express all three isoforms of PFK-1. In breast cancer, PFKP, but not PFKM or PFKL, correlates with malignant features and poor survival [36,37,38,39]. Unlike PFK-1, PFK-2 catalyzes the production of fructose-2, 6-bisphosphate (Fru-2,6-P2) from fructose-6-phosphate (Figure 2). Although PFK-2 does not directly catalyze glycolysis, the product of its enzymatic activity, Fru-2,6-P2, activates PFK-1, making PFK-2 an important regulator of glycolysis [40]. PFK-2 has four isozymes, PFKFB1, PFKFB2, PFKFB3, and PFKFB4. Among these isoforms, PFKFB3 expression is significantly elevated in breast cancer cells [41] and correlates with poor clinical outcomes [42].

Similar to HK, PFK plays important roles in regulating breast cancer glucose metabolism, tumor progression, and drug resistance. Both KLF4 and HIF-1α can activate PFKP gene transcription in breast cancer [43,44]. Under hypoxic conditions, breast cancer cells accumulate citrate through upregulation of PFKP and citrate synthase, and cytoplasmic citrate promotes breast cancer cell migration, invasion, and metastasis [44]. As a regulator of glycolysis, PFK-2 also has functional roles in breast cancer. For instance, O’Malley and colleagues showed that PFKFB4 phosphorylates and activates steroid receptor coactivator-3 (SRC-3), switching glucose flux to the pentose phosphate pathway and promoting purine synthesis by upregulating transketolase [45]. Notably, shRNA-mediated knockdown of SRC-3 or PFKFB4 suppressed breast tumor growth and lung metastasis in xenograft models [45]. PFKFB3 has been found to promote breast cancer cell survival under mitotic arrest conditions. Mechanistically, prolonged mitotic arrest activates AMPK, which phosphorylates and activates PFKFB3, resulting in the switch of breast cancer cells from oxidative respiration to glycolysis and sustained cell survival. Inhibition of AMPK or PFKFB3 can sensitize breast cancer cells to microtubule poisons [46]. Metastatic dormant breast cancer cells are typically autophagy^high^PFKFB3^low^, and inhibition of autophagy in these cells induces metastatic reactivation by upregulating PFKFB3 [42]. In addition, PFK-2 has been reported to promote drug resistance in breast cancer. For example, PIM2 mediates phosphorylation and stabilization of PFKFB3, leading to enhanced glycolysis and paclitaxel resistance in breast cancer cells [47]. Moreover, knockdown of PFKFB2 in p53 wild-type breast cancer cells enhanced paclitaxel sensitivity [48], and inhibition of PFKFB3 and PFKFB4 sensitized ER-positive breast cancer cells to ER-targeting therapies [49]. Therefore, targeting PFKFBs represents a therapeutic strategy for treating metastatic breast cancer and overcoming drug resistance.

### 3.3. Pyruvate Kinase (PK)

PK, the enzyme responsible for the third rate-limiting step of glycolysis, converts phosphoenolpyruvate (PEP) to pyruvate (Figure 2). Among the four PK isoforms (PKML, PKMR, PKM1, and PKM2), PKM2 is frequently overexpressed in cancer cells [50]. High PKM2 expression correlates with poor clinical outcomes in breast cancer and several other cancers [51] and is a poor prognosis marker for patients who have received chemotherapy [52]. Moreover, PKM2 was found to be upregulated in tamoxifen-resistant breast cancer cells, and depletion of PKM2 reversed tamoxifen resistance of breast cancer cells through a PKM2-c-MYC-Survivin pathway [53]. In addition, knockdown of PKM2 sensitized breast cancer cells to doxorubicin in vitro and in vivo [54]. However, ablation of *Pkm2* in mouse models of breast cancer did not inhibit tumor progression [55].

Because the activity of PKM2 is regulated by multiple metabolites, oligomerization status, and post-translational modifications [56,57], the function of PKM2 in cancer is likely to depend on the cellular context and the tumor microenvironment. It has been shown that glycerol phosphate mutase 1 can convert PEP to pyruvate to bypass conventional glycolysis regardless of the PKM2 status [58]. In addition, oncogenic kinases, such as ErbB2, phosphorylate PKM2 at Tyr105, leading to the degradation of LATS1 and nuclear translocation of YAP, which enhances tumor growth [59]. Moreover, acetylated PKM2 promotes breast cancer cell proliferation and correlates with breast cancer recurrence [60]. Furthermore, co-activator-associated arginine methyltransferase 1 (CARM1) activates aerobic glycolysis to promote breast tumorigenesis by methylating PKM2, and inhibition of PKM2 methylation disrupts cancer cell metabolic reprogramming and inhibits metastasis [61]. Interestingly, pre-metastatic niche cells can take up tumor cell-secreted miRNA, miR-122, leading to downregulation of PKM2 and decreased glucose uptake in niche cells. Consequently, this tumor-induced metabolic reprogramming of the pre-metastatic niche increases nutrient availability to metastasizing cancer cells and promotes breast cancer metastasis [62].

### 3.4. Pyruvate Dehydrogenase Kinase (PDK)

PDK inactivates pyruvate dehydrogenase by phosphorylating it. Pyruvate dehydrogenase is the primary component of the pyruvate dehydrogenase complex (PDC) (Figure 2). The inactivation of pyruvate dehydrogenase leads to decreased pyruvate oxidation in mitochondria and increased conversion of pyruvate to lactate [63]. Perturbation of the PDC–PDK axis causes a cellular shift from oxidative phosphorylation to glycolysis, permitting cell proliferation under hypoxic conditions [64,65]. PDK1 is required for breast cancer cells to adapt to hypoxia and a nutrient-deprived microenvironment. Notably, liver-metastatic breast cancer cells have high HIF-1α activity, which induces PDK1 expression and glycolysis, and knockdown of HIF-1α or PDK1 inhibits breast cancer liver metastasis [66]. In addition, through kinome-wide RNAi screening, Arteaga and colleagues revealed that the PI3K-PDK1 pathway is involved in acquired resistance to CDK4/6 inhibitors in ER-positive breast cancer [67].

### 3.5. 3-phosphoglycerate Dehydrogenase (PHGDH)

PHGDH is the key enzyme that couples the glycolytic pathway to the serine synthesis pathway by converting 3-phosphoglycerate (3-PG) to 3-phosphohydroxypyruvate (Figure 2) [68]. PHGDH competes with phosphoglycerate mutase for 3-PG, thereby increasing the flux of serine biosynthesis and activating downstream metabolic enzymes of the serine synthesis pathway, which provides cancer cells with α-ketoglutaric acid and folate for growth and proliferation. Although a number of reports indicate that PHGDH plays a key role in breast cancer tumorigenesis, progression, and metastasis, the conclusions remain controversial. In breast cancer, higher PHGDH expression is associated with higher tumor grade and worse survival [69]. In an in vivo shRNA screen using the MCF10CDIS.COM human breast cancer cell line, PHGDH was identified as a key player in breast tumorigenesis [70]. Moreover, depletion of PHGDH in the TNBC cell line Hs578T inhibited tumor growth [71]. However, a study from Novartis showed that knockdown of PHGDH in two TNBC cell lines, HCC1806 and BT-20, did not affect tumor growth in xenograft models [72].

Similarly, conflicting results on the role of PHGDH in breast cancer metastasis have been reported. Semenza and colleagues reported that knockdown of PHGDH in the MDA-MB-231 human breast cancer cell line increased the sensitivity to chemotherapeutic drugs and suppressed lung-metastatic ability [73]. By using the brain-metastatic subline of MDA-MB-231 cells and an intracardiac injection-based brain metastasis model, Pacold and colleagues demonstrated that PHGDH is required for breast cancer brain metastasis [74]. While genetic depletion or pharmacologic inhibition of PHGDH blocked brain metastasis, overexpression of active PHGDH, but not the catalytically inactive mutant, promoted brain metastasis formation [74]. However, a recent study by Fendt and colleagues showed that the presence of PHGDH^low^ cells in human triple-negative breast tumors is associated with cancer cell dissemination and metastasis [75]. In a 4T1 mouse mammary tumor model, both circulating tumor cells and early metastatic lesions were enriched in PHGDH^low^ cells, and knockdown of PHGDH promoted lung metastasis [75]. Because different models were used in these studies, it is likely that the role of PHGDH in breast cancer depends on the genetic background, cellular context, and microenvironment, which warrants further investigation.

### 3.6. Lactate Dehydrogenase (LDH)

LDH is the last enzyme in glycolysis, which catalyzes the conversion between pyruvate and lactate (Figure 2). Among different LDH isoforms, lactate dehydrogenase A (LDHA) is overexpressed in breast cancer cells and facilitates the glycolytic process by converting pyruvate to lactate [76,77]. Knockdown of LDHA in ErbB2-transformed mouse mammary tumor cells inhibited their tumorigenicity [77], suggesting that ErbB2 induces mammary tumorigenesis through, at least in part, LDHA. Subsequent work revealed that in ErbB2-amplified human breast cancer cells, ErbB2 activates HSF1, which in turn binds to the LDHA gene promoter to induce LDHA expression and promote glycolysis and growth [78]. Moreover, LDHA has been reported to promote glycolysis and autophagy in ER-positive, tamoxifen-resistant human breast cancer cells, and LDHA knockdown or inhibition (by oxamate treatment) in these cells restored the sensitivity to tamoxifen [79]. In addition, a recent study revealed that epinephrine, induced by chronic stress, promotes breast cancer stem-like traits through LDHA-dependent metabolic rewiring [80]. Mechanistically, epinephrine activates LDHA to produce lactate, and the pH adjustment promotes USP28-dependent deubiquitination and stabilization of MYC, which in turn activates genes (such as SLUG) involved in breast cancer stem-like properties [80]. Collectively, targeting LDHA represents a promising strategy for overcoming tamoxifen resistance and cancer stemness.

## 4. Glucose Metabolism Reprogramming in the Breast Tumor Microenvironment (TME)

**Figure 3:**
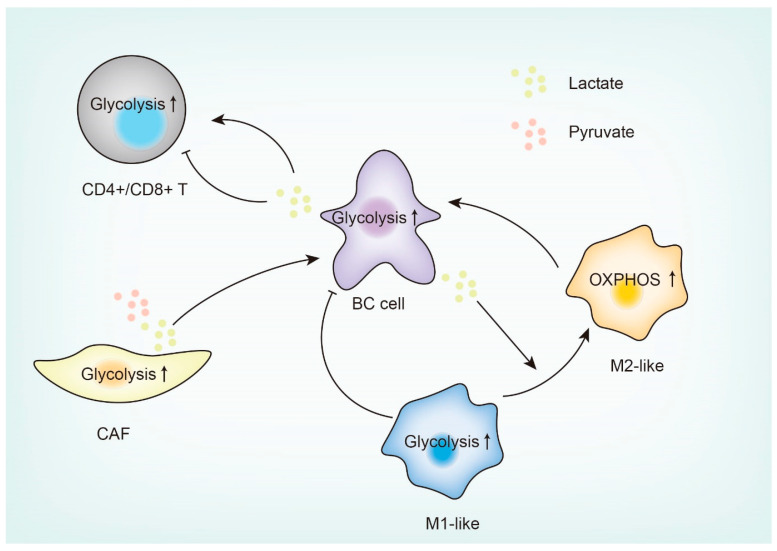
Glucose metabolic crosstalk between breast cancer cells and the tumor microenvironment. Glucose metabolic crosstalk between breast cancer (BC) cells and tumor-associated macrophages (TAMs), tumor-infiltrating T cells (TILs), or cancer-associated fibroblasts (CAFs) is mediated by glucose metabolites such as lactate and pyruvate. Lactate can contribute to the transition of macrophages from M1-like to M2-like, while acting as a double-edged sword in modulating T cells. CAFs undergo a reverse Warburg effect, producing lactate and pyruvate which are taken up by breast cancer cells to promote their proliferation. The upward arrow indicates increased activity in the specific metabolic pathway–glycolysis or oxidative phosphorylation (OXPHOS).

Reprogramming of glucose metabolism occurs not only in cancer cells but also in tumor-associated stromal cells. Metabolic crosstalk between cancer cells and stromal cells promotes tumor progression, metastasis, and therapy resistance [81,82,83]. Here, we discuss glucose metabolism reprogramming in three well-studied tumor-associated stromal cells: macrophages, T cells, and fibroblasts (Figure 3).

### 4.1. Tumor-Associated Macrophages (TAMs)

Depending on the stimuli, macrophages can polarize into either the anti-tumor subtype (M1) or the pro-tumor subtype (M2). M1 macrophages recognize and kill cancer cells by secreting inflammatory factors and through cytotoxic T cell (CTL)-mediated cell-killing effects, while M2 macrophages promote tumor growth and progression by secreting anti-inflammatory factors and by suppressing antitumor immunity (Figure 3) [84]. M1 macrophages have a higher glycolytic flux, while M2 macrophages tend to undergo OXPHOS [85]. Tumor-associated macrophages (TAMs) are usually enriched in a hypoxic microenvironment and are polarized into the M2 phenotype [86]. Hypoxic TAMs have been reported to upregulate a negative regulator of mTOR, named regulation development and DNA damage response 1 (REDD1), leading to inhibition of mTOR, glucose uptake, and glycolysis, which in turn induces the formation of abnormal blood vessels and metastasis [87]. A subsequent study [88] indicates that M2-like TAMs have a high capacity for glucose uptake, which elevates intracellular O-GlcNAcylation levels to promote chemoresistance and metastasis. Mechanistically, O-GlcNAc transferase (OGT) mediates the O-GlcNAcylation of the protease cathepsin B and promotes the secretion of mature cathepsin B into the TME [88]. Although both reports concluded that M2-like TAMs promote cancer, the mechanisms are distinct. Although both studies showed that M2-type TAMs have high GLUT1 expression, neither study determined the loss-of-function effect of GLUT1. In addition, it is worth noting that lactate can contribute to TAM polarization. The lactate produced by tumor cells is a byproduct of glycolysis and is often secreted into the TME. It has been shown that lactate induces M2-like polarization of macrophages, promoting tumor invasion and metastasis [89,90,91].

### 4.2. Tumor-Infiltrating T Cells (TILs)

The metabolic interplay between T cells and cancer cells has an important role in immunotherapy resistance. Whereas regulatory T cells (Tregs) are oxidative and have low glycolysis rates, effector T cells upregulate glycolysis and glutamine metabolic pathways to promote proliferation and differentiation (Figure 3) [92,93,94]. High levels of lactate in the TME inhibit the activity of CD4^+^ T cells and CD8^+^ T cells due to the depletion of intracellular NAD by lactate [95,96]. On the other hand, Tregs are not inhibited by lactate, and they maintain intracellular NAD levels mainly through mitochondrial metabolism [97]. This could explain why TNBC with high levels of glycolysis responds poorly to immune checkpoint inhibitors (ICIs). Thus, targeting LDH may enhance immunotherapy response. Indeed, Shao and colleagues found that LDH inhibition boosted tumor response to anti-PD-1 in metabolic-pathway-based subtype 2 TNBC, a glycolytic subtype of TNBC [98]. Moreover, depletion of LDHA in mouse mammary tumors enhanced the therapeutic activity of the CTLA-4 blockade [99]. In addition, ablation of monocarboxylate transporter receptor 1 (MCT1, a lactate transporter) in Tregs could reverse immunosuppression in the lactate-rich TME and improve immunotherapy efficacy [100]. In a mouse model of breast cancer, treatment with an MCT1/MCT4 inhibitor improved the efficacy of ICIs (anti-PD1 plus anti-CTLA-4) [101]. On the other hand, however, excise-elevated lactate enhanced the cytotoxicity of CD8^+^ T cells and inhibited tumor growth, suggesting that moderately elevated lactate might promote anti-tumor immunity [102], although the molecular mechanism remains unclear.

### 4.3. Cancer-Associated Fibroblasts (CAFs)

Influenced by tumor cells, CAFs undergo a remarkable metabolic switch from oxidative phosphorylation to aerobic glycolysis in the TME, and the “reverse Warburg effect” theory was proposed to describe this phenomenon (Figure 3) [12]. Mechanistically, hypoxia in the breast TME enables epigenetic reprogramming of pro-glycolytic genes, resulting in higher glycolytic flux [103]. The reverse Warburg effect is associated with multi-drug resistance of breast cancer cells, but how tumor cells educate CAFs to activate drug resistance-related pathways remains unclear [104]. CAFs are heterogeneous and display different molecular characteristics and functions. Mechta-Grigoriou and colleagues identified four CAF subgroups (S1–S4) in breast cancer that are differentially enriched in different breast cancer subtypes [105]. Caveolin 1, one of the CAF markers, stimulates glycolytic flux and secretion of lactate, pyruvate, and glutamine into the TME. Adjacent breast cancer cells take up these metabolites to promote their proliferation and stemness [106]. In addition, a CAF subpopulation characterized by CD10 and GPR77 expression supports breast cancer stemness and chemoresistance [107]. The emerging single-cell technologies may enable further identification of CAF subpopulations based on metabolic characteristics.

### 4.4. Other Immune Cells in the TME

In addition to the three extensively studied cell types discussed above, natural killer cells and dendritic cells are also influenced by the reprogramming of glucose metabolism in breast cancer cells. The accumulation of lactic acid in the TME has been shown to exert a profound effect on the growth and functions of these two immune cell types. For instance, in syngeneic immunocompetent breast cancer and melanoma models, knockdown of lactate dehydrogenase A (LDHA) in tumor cells reduced lactate production and normalized the acidic TME, which facilitated the infiltration of tumors by natural killer cells and CD8^+^ T cells, leading to inhibition of tumor growth [108]. Conversely, excessive lactate accumulation in the TME attenuates IFNα production by plasmacytoid dendritic cells [109]. In addition, lactate boosts tryptophan metabolism and kynurenine production by plasmacytoid dendritic cells, which contributes to the induction of FoxP3^+^ CD4^+^ regulatory T cells, ultimately promoting tumor progression [109]. Thus, an oncometabolite resulting from glucose metabolic reprogramming in cancer cells can drive pro-cancer reprogramming of tumor-associated dendritic cells.

## 5. Targeting Glucose Metabolism to Improve Cancer Therapy

Since glucose metabolic reprogramming plays important roles in breast cancer metastasis and drug resistance, targeting key enzymes in glucose metabolism pathways holds the potential to prevent or treat metastases and overcome therapy resistance. Although no metabolic drugs have been approved by the FDA to treat breast cancer yet, several inhibitors of glucose metabolism signaling pathways are currently being investigated in preclinical and clinical phases (Table 2) [110]. Thus far, none of these inhibitors have shown satisfactory results when used as single agents.

Lonidamine (LND) is an inhibitor targeting several key players in metabolic pathways, such as hexokinase II (HKII), respiratory chain complex I/II, and monocarboxylate transporter (MCT) [111]. When used as a single agent, the responses to LND were limited and reversible in clinical trials and the side effects were strong at high doses [111,112,113]. Because the side effects of LND do not overlap with the main side effects of chemotherapeutic agents, LND has become a candidate to improve chemotherapy sensitivity. In a randomized multicenter trial, the epirubicin plus LND regimen showed significantly better response rates than epirubicin alone (60.0% vs. 39.8%; *p* < 0.01) [114]. However, in another clinical trial of epirubicin combined with LND for treating metastatic breast cancer, there was no significant difference in overall survival between the combination therapy group (epirubicin + LND) and the epirubicin group [115]. One possible explanation is the difference in the dose of LND used in these two clinical trials (600 mg daily vs. 450 mg daily). Nevertheless, in another randomized multicenter trial to treat breast cancer patients with liver metastases, LND significantly improved the patient response to doxorubicin: while the doxorubicin group had a 33% response rate, the combination therapy group (doxorubicin + LND) showed a response rate of 68% (*p* = 0.03) [116]. The side effects of LND (mainly liver toxicity) due to the high dosages during cancer treatment limit its clinical application, and it may be beneficial to develop methods for tumor-specific delivery of LND.

3-bromopyruvate (3-BrPA), another anti-glycolytic agent, targets multiple players in the glucose metabolism pathway, including HKII, 3-PGK, and GAPDH [117,118]. 3-BrPA has been shown to inhibit tumor growth, angiogenesis, and metastasis [118]. Moreover, it enhances cancer cell chemosensitivity by inactivating ATP-binding cassette (ABC) transporters, suggesting the potential value of 3-BrPA in combination therapy [119,120]. Because of the toxicity of 3-BrPA, it has been suggested that unformulated 3-BrPA should not be used in the clinic, and that formulating 3-BrPA into vehicles such as nanoparticles will not only improve the anti-cancer efficacy but also reduce the side effect [110,121].

Increased uptake of glucose in breast cancer cells and other cancer cells is associated with overexpression of glucose transporters (GLUTs), primarily GLUT1 [122,123,124]. Consistently, GLUT1 is critical for glucose uptake by breast cancer cells [125]; thus, a direct therapeutic strategy is to block GLUT1-mediated glucose transport. Several GLUT1 inhibitors, such as STF-31, WZB117, WZB27, WZB115, and BAY-876, have been developed and tested in preclinical models, and these compounds were found to inhibit glucose uptake and/or glycolysis, suppress cell proliferation, and induce apoptosis in multiple breast cancer cell lines [126,127,128]. In addition, in preclinical breast cancer models, GLUT1 inhibitors have shown synergistic effects with current cancer therapies, including radiation treatment, cisplatin, paclitaxel, and adriamycin [129,130,131]. It should be noted that these GLUT1 inhibitors have not been advanced to the clinic. One concern is that GLUT1 protein is ubiquitously expressed in nearly all mammalian tissues and is highly abundant in the brain and erythrocytes [132]. Thus, tumor-specific inhibition of GLUT1 warrants future investigation.

In addition to the therapeutic potential, the diagnostic accuracy of breast imaging with positron emission tomography (PET) has been investigated, given that breast cancer is often characterized by elevated uptake of the glucose analog, radiolabeled fluorodeoxyglucose (FDG) [133,134,135,136]. PET has shown high accuracy in detecting breast tumors >2 cm and encouraging results in identifying regional lymph node metastases [137]. In one clinical study [138], Avril and colleagues reported positive correlations of FDG uptake with histologic type, microscopic tumor growth pattern, and tumor cell proliferation; however, they found no significant correlation between FDG uptake and GLUT1 immunoreactivity. In a meta-analysis, Meyer and colleagues reported that GLUT expression levels and standardized uptake values derived from FDG-PET were only moderately associated with various cancers [139]. Of note, approximately half of breast cancer patients do not exhibit overexpression of GLUT1 [140]; whether increased glucose transporter activity of GLUT1 contributes to elevated glucose uptake without upregulation of GLUT1 expression in these patients remains to be determined.

**Table 2:** Anti-glycolytic agents in preclinical and clinical development for cancer therapy.

Druggable Target	Drug	Stage of Development	References
GLUTs	STF-31	Preclinical	[127]
	WZB117, WZB27, WZB115	Preclinical	[126,130]
	BAY-876	Preclinical	[128]
HK2	Silybin	Phase I (NCT00487721)	[141]
	2-DG	Phase I (NCT00096707)	[142]
	lonidamine	Phase I–III	[115]
GAPDH	3-BrPA	Preclinical	[143]
	Koningic acid	Preclinical	[144]
PDK	Dichloroacetate	Phase I (NCT01111097)	[145]
MCTs	AZD3965	Phase I (NCT01791595)	[146]

## 6. Conclusions

Cancer cells rewire glucose metabolism pathways to meet their energy and metabolic needs, which contributes to drug resistance and metastasis. Targeting reprogrammed glucose metabolism in cancer cells represents a potential strategy for improving cancer therapy. Challenges in making such translational advances include the following: (1) Multiple isoforms of metabolic enzymes catalyze the same reaction, and small-molecule inhibitors usually do not distinguish between different isoforms expressed in cancer cells and normal cells, which could cause substantial toxicity or side effects. (2) Cancer-specific glucose metabolic enzymes are usually overexpressed in tumor cells, and thus high concentrations of inhibitors are often required to effectively suppress enzyme activities, which poses human tolerance challenges. (3) Cancer cells exhibit metabolic heterogeneity, with different subtypes of breast cancers (or different cancer cells within the same tumor) exhibiting different metabolic profiles. Thus, it is difficult to develop a broadly applicable metabolic drug as a single-agent therapy for heterogeneous cancers.

Metabolites in the TME have important roles in modulating the anti-cancer or pro-cancer effect of tumor-associated stromal cells. Thus far, most TME metabolite studies are focused on lactate, a major glucose metabolite leading to acidic TME. It is encouraging that inhibitors of lactate signaling have enhanced immunotherapy response in preclinical studies. In the future, the characterization of additional TME metabolites will not only advance our understanding of tumor progression but also shed new light on cancer treatment.

To alleviate systemic toxicities of glucose metabolic inhibitors, it will be helpful to develop tumor-targeting vehicles to carry these drugs, and/or to design prodrugs (inactive forms) that can be converted to active drugs by enzymes specifically expressed in cancer cells. In addition, it will be beneficial to develop combination therapies that combine glucose metabolic inhibitors with other therapies, including chemotherapy, radiotherapy, targeted therapy, and immunotherapy.

## Figures and Tables

**Table 1:** Drug resistance associated with alterations of enzymes in glucose metabolism.

Altered Enzyme	Drug Resistance	Type of Cancer
HK	Tamoxifen	Breast cancer
	Paclitaxel	Breast cancer
	Letrozole	Breast cancer
	Trastuzumab	Lung cancer
PFK	Paclitaxel	Breast cancer
	Cisplatin	Endometrial cancer
PK	Tamoxifen	Breast cancer
PDK	Tamoxifen	Breast cancer
PHGDH	Tamoxifen	Breast cancer
	Doxorubicin	Breast cancer
LDH	Tamoxifen	Breast cancer
